# Exploring carbohydrate binding module fusions and Fab fragments in a cellulose-based lateral flow immunoassay for detection of cystatin C

**DOI:** 10.1038/s41598-022-09454-9

**Published:** 2022-03-31

**Authors:** Satheesh Natarajan, Jayaraj Joseph, Duarte Miguel França Prazeres

**Affiliations:** 1 Healthcare Technology Innovation Centre, Indian Institute of Technology, Madras, Chennai, 600113 India; 2 Department of Electrical Engineering, Indian Institute of Technology, ESB 317, Measurements and Instrumentation Laboratory, Chennai, 600036 India; 3 Department of Bioengineering, iBB- Institute for Bioengineering and Biosciences, Instituto Superior Técnico, Universidade de Lisboa, Av. Rovisco Pais, 1049-001 Lisbon, Portugal; 4 Associate Laboratory i4HB—Institute for Health and Bioeconomy at Instituto Superior Técnico, Universidade de Lisboa, Av. Rovisco Pais, 1049-001 Lisbon, Portugal

**Keywords:** Assay systems, Biochemical assays

## Abstract

This paper presents a lateral flow assay (LFA) for the quantitative, fluorescence-based detection of the kidney biomarker cystatin C that features conjugates of capture antibodies and fusions of carbohydrate binding modules (CBM) with ZZ domains anchored on cellulose deposited over nitrocellulose (NC). The ZZ-CBM3 fusion provides a biomolecular interface between the cellulose layer and the Fc portion of the capture antibodies. By resorting to detection Fab fragments that lack the Fc portion we overcome the observed interference of full-length detection antibodies with the ZZ-CBM3 fusion at the test lines. Using the new LFA architecture, a linear concentration–response relationship was observed in the 0–10 ng/mL cystatin C concentration range, which is compatible with the clinically normal (5–120 ng/mL) and abnormal (> 250 ng/mL) levels of cystatin C, as long as proper dilutions are made. An inter assay CoV of 0.72% was obtained. Finally, mock urine samples characteristic of normal (100 ng/mL) and kidney tubular disease (4000 ng/mL) patients were successfully analyzed. Overall, we demonstrate an innovative LFA architecture that combines NC strips with layered cellulose, ZZ-CBM3 fusions and fluorescently labeled Fab fragments.

## Introduction

Lateral flow assays (LFA) are one of the key players in the Point-Of-Care (POC) testing market. The portability of these devices makes them an excellent solution to perform diagnostics in the context of (1) health emergencies that require fast results for decision making, (2) diagnosis in remote areas, (3) monitoring of patients, (4) testing in primary-care appointments and (5) self-monitoring^1–3^. The current SARS-CoV-2 coronavirus pandemic provides an excellent example of the advantages and complementarity of LFA diagnostics, with numerous tests being extensively used to detect anti-viral antibodies (e.g. IgG, IgM) and SARS-CoV-2 antigens^4–7^. Many other applications of LFA have been reported, including the monitoring of hematology parameters, cholesterol, cortisol, pregnancy and fertility; diagnosis of infectious diseases (e.g. Covid-19, hepatitis C, HIV) and testing of disease markers; activated clotting time and coagulation analysis; and control of drugs-of-abuse, among others^8^. The environmental, veterinary, forensics, agro-food and bio-defense areas have also explored LFA for testing at the point-of-contact^9,10^. The current relevance and impact of LFA in the diagnostics arena could significantly expand if drawbacks like low sensitivity, low specificity, and lack of quantitation can be overcome. Significant research efforts are thus being devoted to bringing the overall performance of LFAs close to that afforded by standard laboratory tests like ELISA and PCR^11^. Innovation avenues being pursued include the use of stacking pad configurations to extend antigen/antibody binding interactions^12^, the integration of sponge shunts to decrease fluid flow rates^13^ and modifications of the standard LFA architecture^14^, or the use of up-converting phosphors reporters^15^ to increase sensitivity and improve detection limits.

Conventional LFA encompass overlapping rectangular strips mounted on a backing card. Key components include a sample pad, a conjugate release pad, an analytical strip and an absorbent pad. These are typically combined with reagents that are specific for the recognition of the target analyte. The individual components in the assay provide support (the backing card), receive the liquid sample (the sample pad), hold test reagents (release pad), harbor test and control lines for signal generation and detection (the analytical strip) and act as a sink for the liquid that runs through the LFA (absorbent pad)^16,17^. This simple design is predominant across the field and has hardly been modified over the years, even though several modifications have been proposed^12–15^.

**Figure 1:**
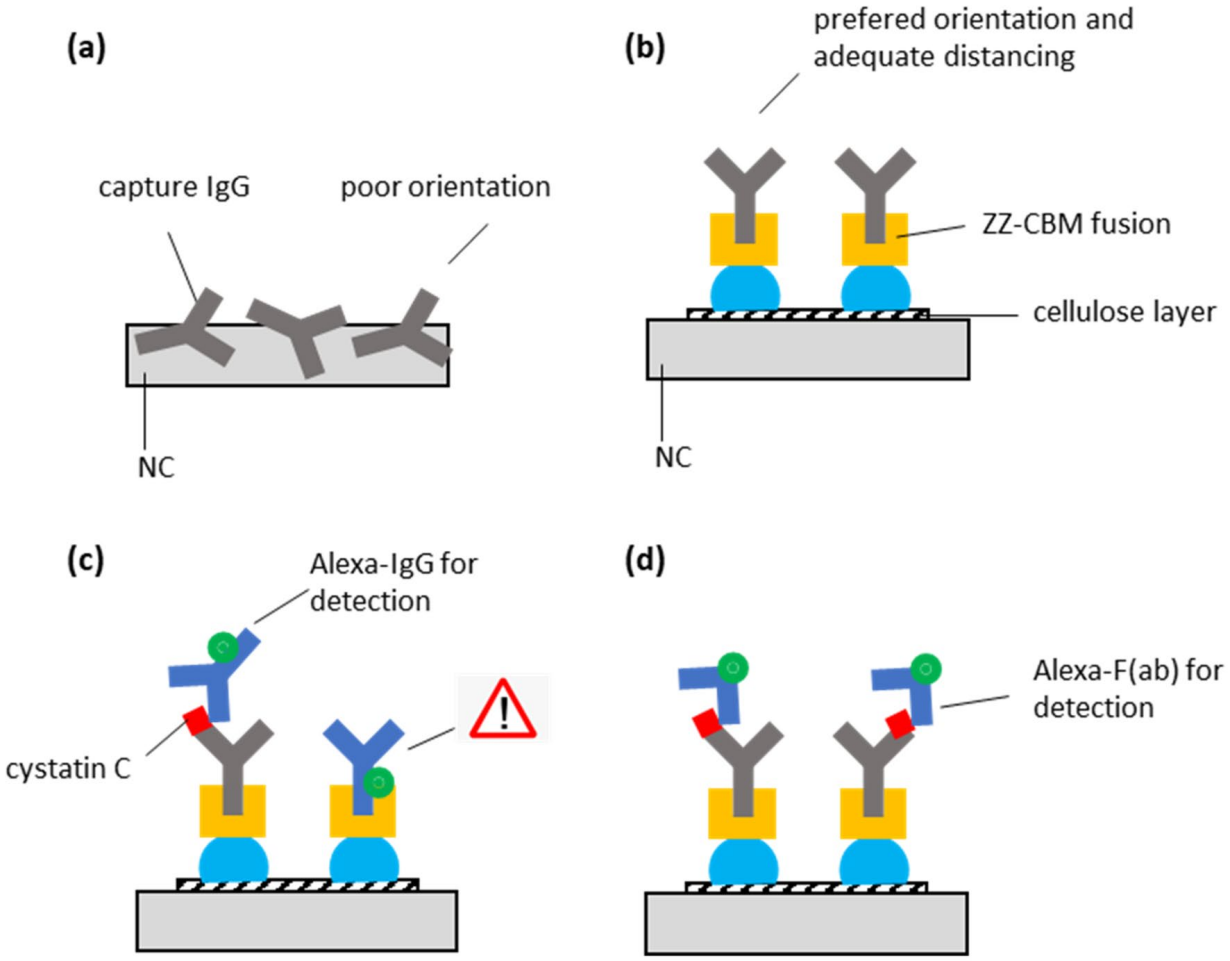
Biomolecular architecture based on ZZ-CBM fusion for the capture and detection of cystatin C on cellulose-based LFA cartridges. (**a**) The traditional LFA format relies on the random adsorption of capture antibodies over the test lines of a NC. This makes it difficult to control the orientation and accessibility of the antibodies, which can take different positions in space after immobilization. (**b**) By introducing a layer of cellulose at the active test line of LFA strips, a ZZ-CBM-based fusion can be used to properly anchor and orient the capture antibodies (CBM binds to cellulose, ZZ captures antibodies via the Fc portion). (**c**) Anti-cystatin C antibodies anchored on strips via ZZ-CBM fusions can capture complexes of cystatin C with Alexa-labeled anti-cystatin detection antibodies. However, this has the problem that ZZ-CBM fusions can capture the detection antibodies and originate non-specific signals in the absence of cystatin C. (**d**) The use of Alexa-labeled Fab fragments, which lack an Fc portion, circumvents the problem highlighted in (**c**).

In most cases the analytical strip in LFA is made from nitrocellulose (NC). Apart from its excellent ability to bind proteins (80–100 μg/cm^2^), other features that make NC ubiquitous in LFA include the ability to move fluids by capillarity, availability at low cost and ease of handling^16,18^. Nevertheless, NC may not be the best matrix for an LFA. For example, a key flaw is linked to the fact that capture molecules (e.g. antibodies) are randomly adsorbed over NC (Fig. 1a)^19^. This lack of suitable orientation of the capture molecules after immobilization ultimately results in a less effective capture of analytes^16^. Several attempts were explored to overcome this limitation and favor proper orientation, which include the development of methods to covalently immobilize antibodies and proteins on NC^20,21^, the use of a NC-binding mutant streptavidin^22^ and the development of NC-binding anchor proteins^23^. Another strategy relies in replacing the NC altogether, for example by introducing analytical strips made of cellulose^24–28^. Apart from being an attractive and popular material for biosensors and LFA^29^, cellulose offers the opportunity to explore the natural affinity of Carbohydrate Binding Module (CBM) towards carbohydrates^30^.

CBMs are discrete protein modules characteristic of many carbohydrate-active enzymes. Their ability to bind a diversity of carbohydrates like disaccharides, oligosaccharides and polysaccharides such as cellulose with high selectivity and specificity has originated several biotechnological applications^31,32^. Many of these are based on bifunctional fusions that combine CBM with biomolecular partners to promote the anchoring and capture of biomolecules to cellulose materials^33–36^. For example, fusions that combine CBMs with ZZ domains were designed that bind IgG antibodies via their Fc region. Such ZZ-CBM fusions were used to anchor antibodies to paper microfluidic devices^33^ and to cellulose microparticles for the development of molecular tests^35^. More recently, Yang et al*.* described a construct that was prepared by genetically fusing a CBM3 and a CBM1 with B and C domains of protein A and further used to anchor capture antibodies in test lines of a cellulose-based LFA^27^. When applied to prostate specific antigen detection, the cellulose-LFAs with CBM fusions were found to be sixfold more sensitive than conventional LFAs that used simple physical adsorption of capture antibodies. Nevertheless, the authors highlighted that since CBMs can interact with Fc regions of any IgG, nonspecific binding of the CBM fusion at the test line to detection antibodies could be a significant problem^27^. To resolve this issue, Elter and co-workers proposed the direct fusion of CBM to single chain variable fragments (scFv) or to full-length antibodies^28^. This strategy, however, requires that specific CBM-antibody fusions must be constructed, produced and purified for each different antibody one needs to anchor. By comparison, the use of ZZ-CBM fusions is much more flexible since a single fusion can be used to anchor virtually any Fc-containing immunoglobulin (Fig. 1b).

Despite the promising results, the adoption of cellulose analytical strips in LFA design is unlikely to occur soon given the dominance of NC strips in the field. Apart from the track record and advantages, the fact that handling NC in LFA manufacturing uses very well-established procedures and dedicated machinery^9^ is likely to deter manufacturers from changing to cellulose. One way to counter this could be to incorporate cellulose on a conventional NC strip. This could be accomplished for example by layering controlled amounts of cellulose over the test and control lines of an NC strip. For example, suspensions of cellulose nanofibers (CNF) or solutions of CNF dissolved in NMMO (*N*-methylmorpholine *N*-oxide) can be deposited over the test regions of NC or paper strips. This addition of cellulose fibers to LFA strips has been shown to improve sensitivity to protein and nucleic detection^26,37,38^. The core idea is that the covering of test regions in NC with a mesh of cellulose fibers can increase the amount of capture biomolecules close to the surface of the strips. Results show that the density of selectively bound complexes of the target analytes with the chosen reporter (e.g. gold nanoparticles^37,38^, Alexa fluorophore^26^) effectively increases in the top part of test lines, enhancing the LFA sensitivity. Here we propose that such layers of cellulose fibers over NC strips can be further used as anchoring points for CBM-fusions, which can then be used to properly anchor and orient capture molecules.

Our goal is to explore cellulose fibers and CBM-fusions to develop an innovative lateral flow immunofluorescence assay. As a model system we target the quantitative detection of cystatin C in urine samples using an image-based reader device (see ImageQuant analyser in supplementary material S1). Cystatin-C (Cys-C) is a low molecular weight (Mr 13,359 Da) cysteine proteinase inhibitor that can be used to screen for and monitor kidney dysfunction^39,40^. The urinary concentration of Cys-C in healthy subjects is recorded in the 5–120 ng/mL range^39^. However, concentrations are substantially higher in patients with chronic nephritis (390–1000 ng/mL^39^) or with kidney tubular disease (250–16,000 ng/mL^40^). The availability of a rapid LFA test for POC and personalized detection of Cys-C would complement existing methods such as particle-enhanced turbidimetric immunoassay^41^, nephelometric immunoassay^42^ and ELISA^43^.

In this work, we introduce a layer of cellulose that is prepared from dissolved CNF in the test and control lines of a NC analytical strip of LFA so that ZZ-CBM fusions can be used to effectively anchor Cys-C capture antibodies at the test lines (Fig. 1b). A possible downside of this approach, as described above, is that the antibodies used for Cys-C detection may effectively compete with the anchored capture antibodies for the binding to the ZZ-CBM fusion in the test line (Fig. 1c). One option could be to select detection antibodies that have lower affinity to ZZ fragments. For example, human IgG3 is known to bind less effectively to protein A when compared to IgG1, IgG2 and IgG4^44,45^. However, human IgG3 may be very difficult to find and procure, given that most antibodies commercially available are either of non-human origin or, if human, do not belong to class 3. A more effective alternative could be to replace the full antibodies used for detection by nanobodies or antibody fragments^46^. Since these are devoid of the constant Fc portion, binding to the ZZ part of the fusion will be inexistent (Fig. 1d). In practical terms, antibody fragments could be generated from full antibodies by resorting to commercial enzymatic antibody fragmentation kits that selectively cleave IgG and IgM immunoglobulin molecules^47^.

Here we report a series of experiments that were designed to evaluate the feasibility of using cellulose layered over NC and CBMs in a lateral flow immunofluorescence assay for Cys-C detection. By exploring the highly efficient cellulose/CBM interaction, a new biomolecular architecture is used that will hopefully result in improved capture and recognition of complexes formed by the target Cyc-C and fluorescently labeled antibody fragments. This could translate into a reduction in the amount of capture molecules required per strip, higher sensitivity of the LFA and improved consistency of the results.

## Results

The material and molecular parts of a conventional LFA cartridge were redesigned to explore the affinity of CBM for cellulose as a means of anchoring Cys-C capture antibodies. On the material side, a cellulose zone was incorporated on standard NC strips by layering cellulose over the test and control lines. On the molecular side, ZZ-CBM3 fusions were used at the cellulose zone of the LFA strip. This fusion, which combines a double Z domain from the staphyloccocal protein A with a type A, family III CBM (CBM3) from the cellulosomal-scaffolding protein A from *Clostridium thermocellum*, has been used successfully to anchor antibodies to paper^33,34^, cellulose microparticles^35^ and cellulose hydrogels^36^. The ZZ domain, which is an engineered variant of the domain B of the staphyloccocal protein A, is expected to capture the anti-Cys-C antibodies via their Fc region^33–36^. The CBM3 module, which displays a distinctive planar linear strip of polar and aromatic residues, located in one of the faces of its 9-strand β-sandwich jelly roll structure^48^, is expected to bind strongly and specifically to the cellulose fibers^34–36^. An initial set of experiments was designed to evaluate the feasibility of using cellulose coatings and CBMs in LFAs, which involved dispensing different biomolecules in the test lines of NC and NC + cellulose strips, addition of Alexa-labeled biomolecules to conjugate pads, cartridge assembly and running of samples containing adequate biomolecules.

### Coating NC with cellulose fibers

**Figure 2:**
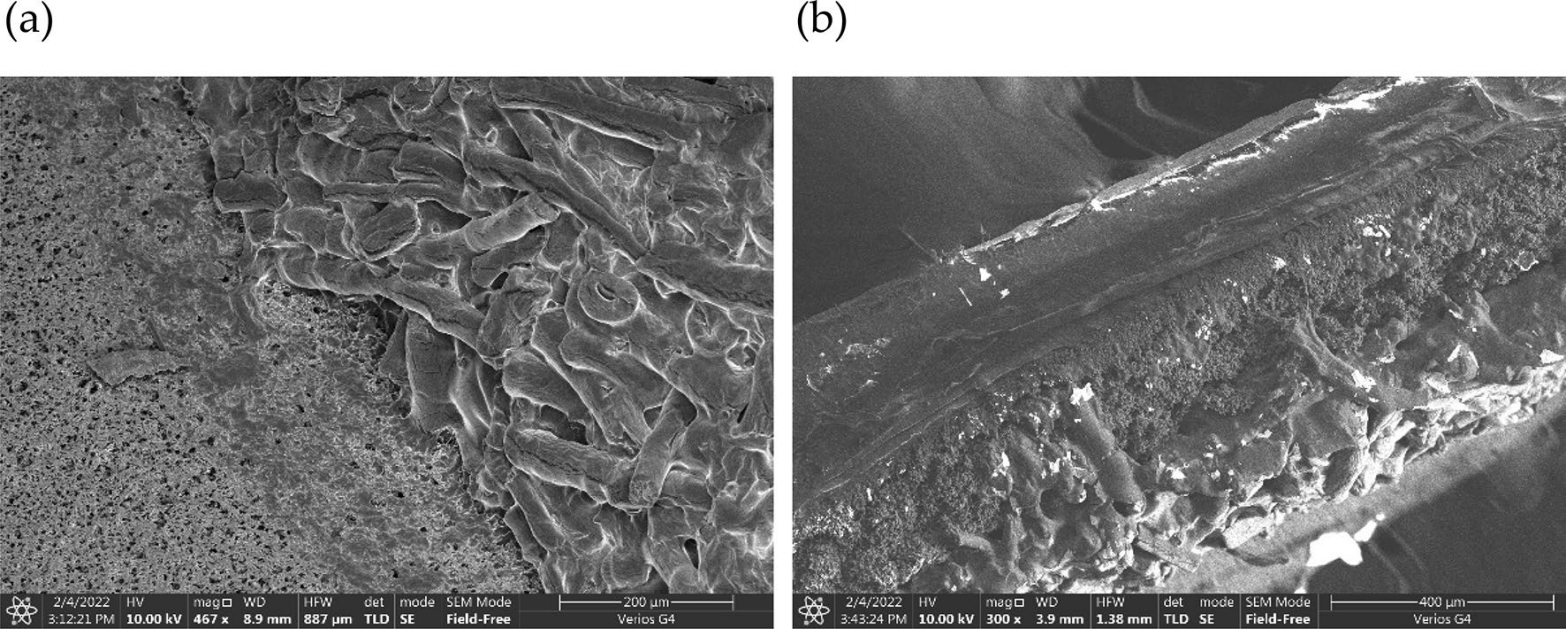
Scanning electron microscopy (SEM) analysis of cellulose fibers regenerated in situ over NC. (**a**) Top view at × 467 magnification and (**b**) cross section at × 300 magnification. The boundary region between NC and cellulose fibers is clearly visible in both images.

Cellulose was incorporated on standard NC strips by dispensing repeatedly (five times) solutions of dissolved cellulose in NMMO over the same position of the test and control zones (see supplementary material S2). During the subsequent process of drying, cellulose fibers form *in-situ* via the bottom-up self-assembly of the dissolved cellulose chains. The final longitudinal width of the cellulose coat on the final NC strip varied between 2.7 and 2.9 mm (see Fig. S2b). The layer of regenerated cellulose fibers over NC was imaged with SEM by analyzing a strip over which cellulose was dispensed (Fig. 2). Images were obtained of the top layer (Fig. 2a) and of cross sections of the NC strip with cellulose (Fig. 2b).

The contrast between the cellulose layer and NC is quite evident in the image of the top view, which captures the boundary region between the two zones (Fig. 2a). The NC region presents the characteristic three-dimensional open pore structure, whereas the cellulose region is covered with a mesh of fibers. These fibers are relatively heterogenous, displaying diameters in the 30–50 µm range and lengths that seldom exceed 300 µm. In some cases, fibers with a hollow interior are visible. A connective layer between some fibers is also apparent in some zones. The cross-section image of the strip further shows that the cellulose layer sits on top of the NC membrane and that the inside structure of NC remains unaltered. Based on these images, we expect that fluid wicking through this region will split between the underlying NC membrane and the top cellulose layer. In other words, part of the fluid by-passes the top cellulose layer. The preparation of regenerated cellulose materials (fibers, films, membranes, hydrogels, etc.) from solutions of cellulose dissolved in NMMO has been described extensively^49,50^. Fiber formation is usually induced by spinning, a process that involves the extrusion of the dissolved cellulose solution through an orifice spinneret and into an air gap, and then regeneration into a coagulation bath^50,51^. Here the fibers regenerate during the process of drying that follows the dispensing, creating a layer of cellulose for affinity interaction with CBM fusions to take place.

**Figure 3:**
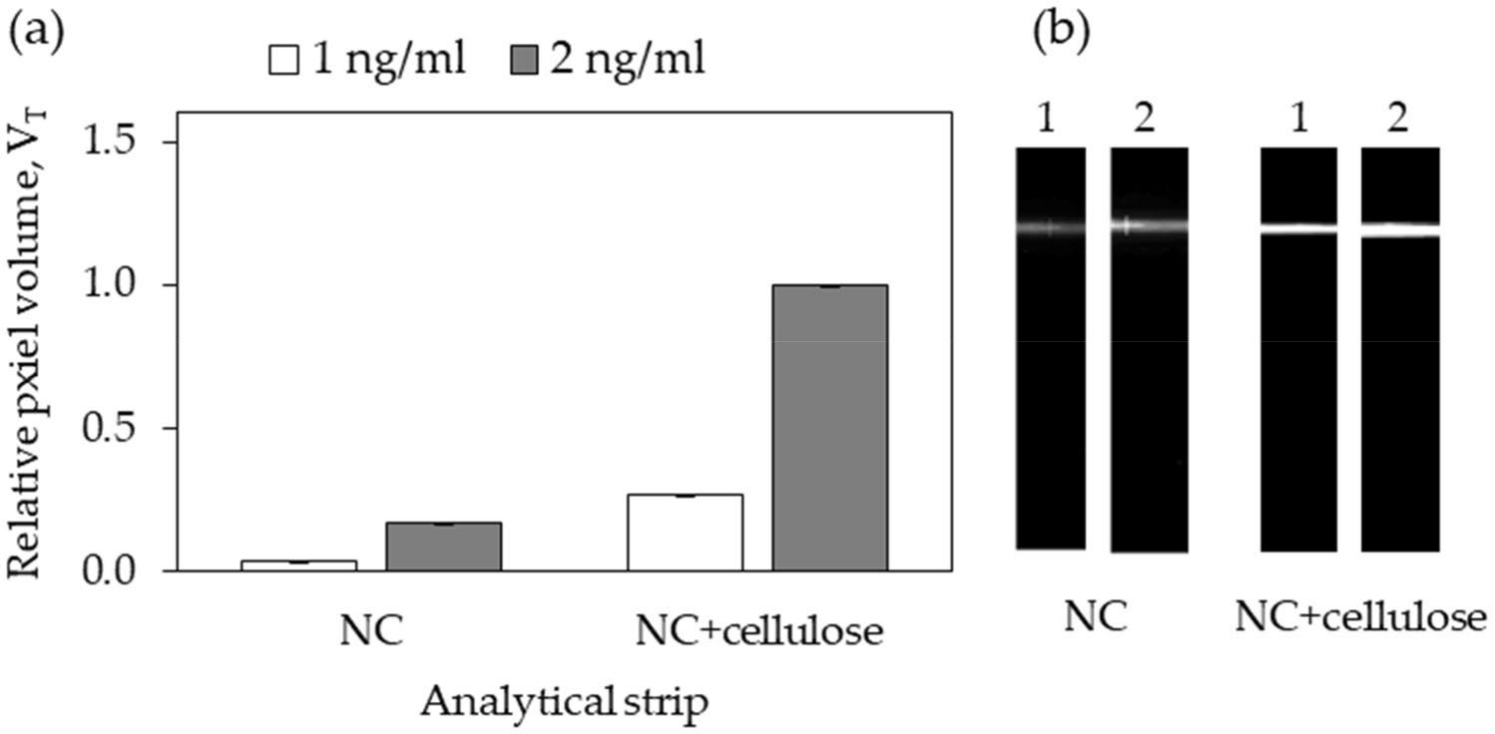
Impact of coating cellulose over NC strips on the fluorescence of signals generated. Lines of biotin-BSA were dispensed on NC and NC + cellulose strips. Following assembly, buffer samples containing either 1 ng/mL or 2 ng/mL of Alexa-labeled streptavidin were run on the cartridges. (**a**) The pixel volume of the lines, V_T_, was obtained and normalized relatively to the highest pixel volume obtained in all experiments. (**b**) Representative black and white fluorescence images of lines in the analytical strips as captured by the ImageQuant camera. Experiments were performed in triplicate.

A set of experiments was performed next using the control system (biotin-BSA:Alexa-streptavidin) to evaluate the impact of the layer of cellulose on the fluorescence of signals generated at the strips. Lines of biotin-BSA were dispensed on NC and NC + cellulose strips and LFA cartridges were assembled. Next, buffer samples containing either 1 ng/mL or 2 ng/mL of Alexa-labeled streptavidin were run. The flowing buffer carried the streptavidin-Alexa conjugates towards the test lines. The pixel volume of the fluorescent lines generated upon capture of the conjugates by the immobilized biotin-BSA was obtained with the ImageQuant analyzer (see details about the calculation of pixel volume of lines in “Materials and methods” section) and normalized relatively to the highest pixel volume (Fig. 3a).

Results show that when cellulose is used as a coating, the fluorescence lines are in general thicker, more intense (see Fig. 3b) and displayed a larger pixel volume (Fig. 3a). The increased thickness of the lines can be partly attributed to an increased lateral diffusion of the biotin-BSA solution on the top cellulose layer upon dispensing. We further suggest that this cellulose layer can adsorb a larger number of biotin-BSA molecules per unit area compared to the plain NC. As a result, the number of fluorescent complexes close to the surface increases, producing more intense signals. Finally, preliminary experiments confirmed that the coat of cellulose effectively constitutes an anchor point for ZZ-CBM3 fusions, as expected (see supplementary material S3).

### Anchoring antibodies in NC strips with a cellulose coat

**Figure 4:**
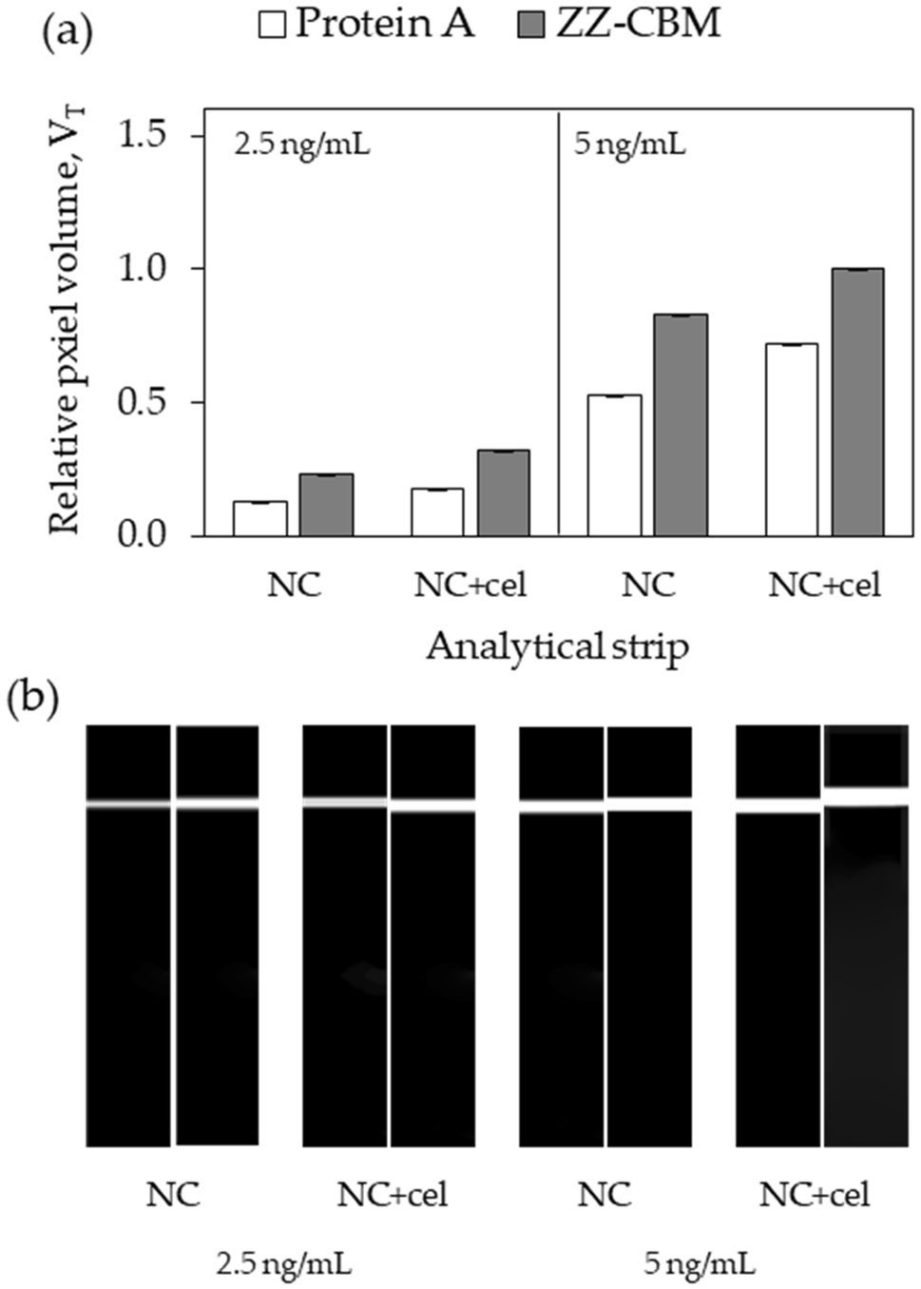
Comparative capture of Alexa-labeled antibodies by protein A and ZZ-CBM3 fusions dispensed on test lines of LFA. Experiments were performed using analytical strips of NC and of NC with a coat of cellulose (NC + cel) on the test line. Samples containing either 2.5 or 5 ng/mL Alexa-antibodies were tested. (**a**) The pixel volume of test lines, V_T_, was normalized relatively to the highest pixel volume obtained in the experiments. (**b**) Representative fluorescence images of the test line region of strips (protein A left, ZZ-CBM3 right) obtained by the ImageQuant camera are shown. Experiments were performed in triplicate.

A first experiment was performed to check if ZZ-CBM3 fusions remain functional after being dispensed on the test line of NC and NC + cellulose strips. For comparative purposes, LFAs were also prepared by dispensing protein A alone on the test lines. The concentration of protein A in the solution used to produce the lines (2 mg/mL) was double the value of the concentration of ZZ-CBM3 solution (1 mg/mL). Since the molecular weight of protein A (64 kDa) is double the molecular weight of ZZ-CBM3 (32 kDa), the lines in the two LFA types had the same molar amounts of either protein. Once assembled, the cartridges were tested with samples containing either 2.5 or 5 ng/mL of Alexa-labeled anti-Cys-C antibodies. The pixel volume of test lines, V_T_, was normalized relatively to the highest pixel volume obtained in the experiments (Fig. 4a). Fluorescence images of the test line region were also captured by the ImageQuant camera (Fig. 4b).

Results show that the immobilized ZZ-CBM3 was able to capture the flowing antibody, whether the fusion was simply adsorbed to a plain NC strip or anchored on the cellulose layer via biomolecular interaction. Moreover, thicker and more intense fluorescence lines were always obtained when the fusion was deposited over the cellulose layer (Fig. 4). This could indicate that more antibodies were captured and/or that there is an increase in sensitivity due to the presence of the cellulose coat, as seen above (Fig. 3) and before^26^. Furthermore, the ZZ part of the fusion that is responsible for the antibody capture is likely to be less hindered by the surface and have a more favorable orientation when it is anchored on cellulose via the CBM3 part, as compared to the situation where it is simply adsorbed to NC.

Control experiments performed with test lines prepared with protein A show that the lines obtained were less intense and narrower when compared with the corresponding lines obtained with ZZ-CBM3 lines (Fig. 4). These results should be interpreted with caution. For once, even though the same molar amounts of protein A and ZZ-CBM3 were used, it should be kept in mind that protein A has in effect five antibody-binding domains (A, B, C, D, and E) compared to the two Z domains of the fusion^52^. Since the affinity towards antibodies is known to vary across these domains, it is virtually impossible to guarantee that the number of antibody capture sites is the same in the two experiments. Additionally, since the D and E domains of protein A have an affinity toward the Fab region of antibodies, the orientation of the capture antibody bound to protein A could be different from that of the antibody bound to CBM-ZZ^52^. Still, we think that the higher intensity observed when ZZ-CBM3 fusions were used to capture antibodies relatively to protein A in NC + cellulose strips can be justified in part by the more favorable orientation of the ZZ domain that is afforded by the fusion (Fig. 1b).

### Interference of detection antibodies with capture antibodies anchored with ZZ-CBM fusions

**Figure 5:**
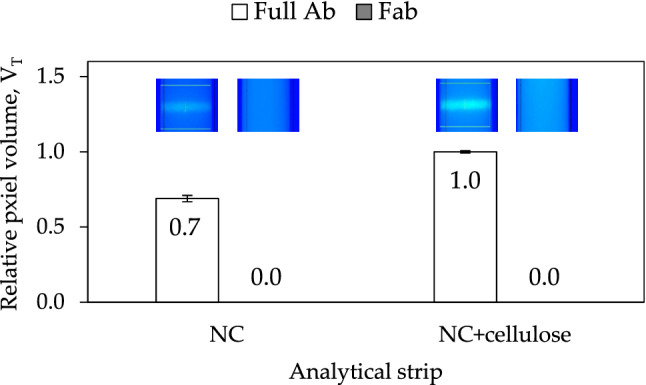
Interference of Alexa-labeled detection antibodies (Full Ab) and Alexa-labeled detection antibody fragments (Fab) with capture antibodies anchored with ZZ-CBM3 fusions on test lines of LFA. Experiments were performed using analytical strips of NC and of NC with a coat of cellulose on the test line. The pixel volume of test lines, V_T_, was normalized relatively to the highest pixel volume obtained. The inset fluorescence images of the test line region were obtained by the ImageQuant camera. Experiments were performed in triplicate.

An experiment was designed next with two specific objectives. Firstly, we wanted to confirm if detection antibodies labeled with Alexa Fluor do compete with capture antibodies when these are anchored on the test line of analytical strips via ZZ-CBM3 fusions (Fig. 1c), as anticipated and also seen by Yang^27^. Next, we wanted to check if this interference issue could be resolved by using Fab fragments of the detection antibody instead of the full-length antibody (Fig. 1d). For this purpose, LFA were assembled with test lines containing conjugates of ZZ-CBM3 and anti-Cys-C capture antibody in the test line, which were then run with samples containing either Alexa-labeled, full-length detection antibodies, or Alexa-labeled Fab fragments of the detection antibodies. As before, tests were made with NC and NC + cellulose strips and the pixel volume of lines in the test region, V_T_, were normalized relatively to the highest pixel volume (Fig. 5).

The results confirmed that the ZZ-CBM3 fusions, albeit being conjugated with the capture antibodies, do indeed capture a fraction of the full-length detection antibodies that flow by, as judged by the appearance of fluorescence in the test lines (see inset images in Fig. 5). This interference is more significant when the ZZ-CBM3:antibody conjugates are anchored on NC + cellulose strips, which further attests to the beneficial role of the cellulose layer seen above. When the full-length detection antibodies were replaced by the corresponding Fab fragments, however, no fluorescence could be observed in the test lines of either the NC and NC + cellulose strips. This result confirms that the use of Fab fragments for detection resolves the issue of interference as anticipated (Fig. 1c and d). Furthermore, additional experiments were performed with LFAs prepared by conventional adsorption of the anti-Cys-C capture antibody in the test line of analytical strips (NC, NC + cellulose) to check if the process used to generate the Fab fragments did not affect their ability to recognize Cys-C. Results confirmed that the Fab fragments were able to bind to Cys-C and generate signals comparable to those generated by full length detection antibodies (see supplementary material S4).

### Detection of cystatin C with capture antibodies anchored with ZZ-CBM3 fusions and Fab detection

A set of experiments was designed next to evaluate the ability of LFAs based on anti-Cys-C capture antibodies anchored with ZZ-CBM3 fusions, and on Alexa-labeled Fab fragments, to detect Cys-C. These experiments were performed using NC strips with a cellulose layer on the test lines. Since we further wanted to check the effect of the amount of capture antibodies in the intensity of signals generated, strips were prepared by dispensing 1:1, 1:2 and 1:4 dilutions of the conjugates of ZZ-CBM3 and anti-Cys-C capture antibody (molar ratio of 1:1.5, 0.5 mg/mL antibody). Conjugate pads were prepared with Alexa-labeled Fab fragments. Following assembly of the LFA cartridges, 10 ng/mL Cys-C samples were run.

**Figure 6:**
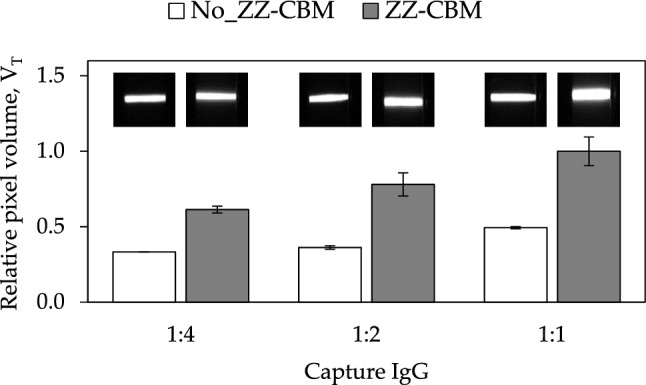
Detection of Cys-C in LFA with capture antibodies anchored on test lines via ZZ-CBM3 fusions and Alexa-labeled Fab. Experiments were performed using analytical strips of NC with a coat of cellulose on the test and control lines. Strips were prepared by dispensing 1:1, 1:2 and 1:4 dilutions of ZZ-CBM3:capture antibody (molar ratio of 1:1.5, 0.5 mg/mL antibody). Controls were also run using LFA with adsorbed capture antibody in the test lines. The pixel volume of test lines, V_T_, was normalized relatively to the highest pixel volume obtained and plotted as a function of the antibody dilution used. Experiments were performed in triplicate. The inset fluorescence images of the test line region were obtained with the ImageQuant camera.

The results confirm that the use of conjugates of ZZ-CBM3 fusions with the capture antibodies in the test lines generates fluorescence signals that are superior to those obtained when adsorbed capture antibodies are used (Fig. 6). As expected, the use of larger amounts of capture antibodies led to an increase in the intensity of the fluorescence signals (Fig. 6). This is also seen by direct observation of the fluorescence images captured by the ImageQuant camera. The images show that lines obtained with ZZ-CBM3 fusions have a thickness that increases with the use of larger amounts of capture antibodies (see inset in Fig. 6).

### Calibration curves

Sets of experiments were performed by running Cys-C standards with various concentrations in the range 0–10 ng/mL in LFA cartridges with the new architecture—analytical strip made of NC with layered cellulose, anti-Cys-C capture antibodies anchored via ZZ-CBM3 and detection with Alexa-labeled Fab fragments (see Fig. S5a in supplementary material S5). An intermediate architecture was also tested that featured analytical strips made of NC alone, anti-Cys-C capture antibodies anchored via ZZ-CBM3 and detection with Alexa-labeled Fab fragments (see Fig. S5b in supplementary material S5). For comparison purposes, the same Cys-C standards were run in LFA cartridges with the conventional LFA architecture—analytical strip made of NC, anti-Cys-C capture antibodies adsorbed on test line and detection with Alexa-labeled full-length antibodies (see Fig. S5c in supplementary material S5). Experiments were performed in triplicate. Representative fluorescence images of the analytical strips in LFA cartridges are shown in supplementary material S6. Well defined test and control fluorescent lines were obtained in all strips. As seen before (see Figs. 3 and 4), the lines were always thicker when a cellulose layer was included in the test and control zones.

**Figure 7:**
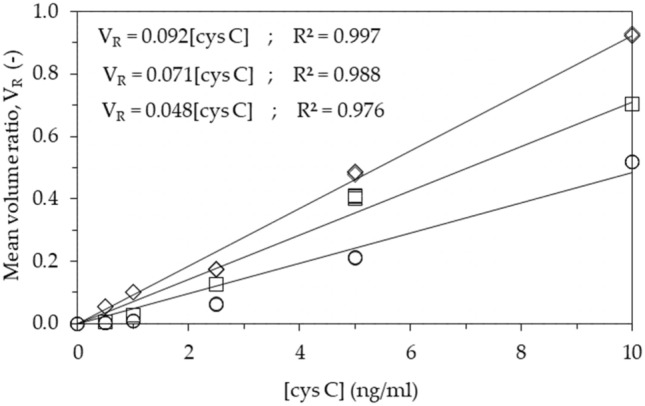
Calibration curve for the detection of Cys-C using LFA cartridges that feature Alexa-labeled Fab fragments and anti-Cys-C capture antibodies anchored via ZZ-CBM3 on analytical strips made of NC with layered cellulose (◇) and NC alone (□). For comparison purposes, the same standards were run in LFA cartridges with the conventional LFA architecture (○)—analytical strip made of NC, anti-Cys-C capture antibodies adsorbed on test line and detection with Alexa-labeled full-length antibodies. The ratio of pixel volume of test line and control line (V_R_ = V_T_/V_C_) is plotted as a function of the concentration of Cys-C ([cys C]). The experiments were run in triplicate and data were fitted by linear regression. Regression equations are shown for the new, intermediate and conventional architecture (top to bottom).

The pixel volume of test (V_T_) and control (V_C_) lines was obtained with the ImageQuant instrument and the response of the LFA devices was measured by calculating the pixel volume ratio, V_R_ (V_T_/V_C_) (see “Materials and methods”). This triplicate V_R_ data was further used to compute the individual CoV, which were then averaged to yield the intra-assay CoV. The values of average CoV of 0.72%, 1.05% and 1.44% were obtained for the new, intermediate and conventional LFA architecture, respectively. This provides a good indication that measurements of Cys-C concentration in the devices are reliable and consistent. Calibration curves were constructed next by plotting the replicate V_R_ data as a function of Cys-C concentration for the new and standard LFA architectures (Fig. 7). A linear behavior of the concentration–response relationship was observed in both cases (see regression statistics data in Fig. 7). The working range obtained for the three LFA is clearly compatible with the clinical diagnostic range for Cys-C (5–120 ng/mL in healthy patients, > 250 ng/mL in patients with kidney disease)^39^, if proper dilutions are made.

### Detection of cystatin C in mock urine samples

The quantitative range for Cys-C concentration afforded by the LFA (up to 10 ng/mL) indicates that urine samples must be diluted when testing healthy (5–120 ng/mL) and diseased individuals (> 250 ng/mL). Experiments were thus designed to detect Cys-C in mock urine samples prepared in artificial urine that are representative of normal patients (100 ng/mL) and kidney tubular disease patients (4000 ng/mL). As a control, and to see if urine components somehow interfere with detection, standard samples with the same concentration were also prepared in buffer. The samples with normal and abnormal Cys-C concentration were diluted with buffer 1:20 and 1:800, respectively, to bring the Cys-C concentration down to 5 ng/mL, which falls within the testing range (up to 10 ng/mL). These diluted samples were then run in LFA cartridges with the new architecture—analytical strip made of NC with layered cellulose, anti-Cys-C capture antibodies anchored via ZZ-CBM3 and detection with Alexa-labeled Fab fragments. Fluorescence images of the corresponding analytical strips are shown in supplementary material S7.

**Figure 8:**
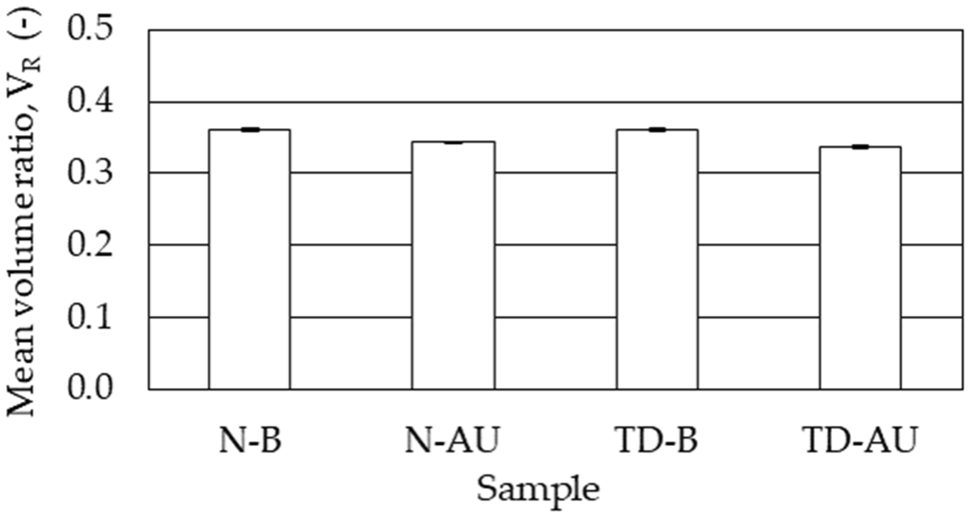
Detection of Cys-C in mock urine samples prepared in artificial urine using the new LFA architecture. Samples that are representative of normal (N) patients (100 ng/mL) and kidney tubular disease (TD) patients (4000 ng/mL) were prepared either in artificial urine (AU) or in buffer (B). The samples were diluted 1:20 (N) or 1:800 (TD) with buffer to bring Cys-C concentration down to 5 ng/mL prior to analysis using LFA cartridges with the new architecture—analytical strip made of NC with layered cellulose, anti-Cys-C capture antibodies anchored via ZZ-CBM3 and detection with Alexa-labeled Fab fragments. The ratio of pixel volume of test line and control line, V_R_, is shown. The experiments were run in triplicate.

The intensity of the fluorescent signals generated at the test line was slightly lower when samples were prepared in artificial urine as compared to buffer (Fig. 8). This could indicate that some components of urine may interfere with the signals generated. If this is in fact the case, the dilution required can be seen as advantageous given that it will dilute out possible interferents. The intensity of fluorescent signals obtained with samples containing abnormal values of Cys-C were equivalent to those obtained with samples representative of normal patients. This was expected since the dilution factors were selected to produce samples with the exact same Cys-C concentration.

## Discussion

Lateral flow assays are widely used in biomedical diagnostics, food contaminant and toxic chemical detection, and environmental monitoring^11^. Their relevance and impact as point-of-care devices has been amply demonstrated with the current SARS-CoV-2 coronavirus pandemic^4–7^. However, and despite their usefulness, drawbacks like low sensitivity, low specificity, and lack of quantitation are often associated with LFAs^11^. Efforts to improve the performance of LFA are on-going, which include the design of new architectures^12–14^, the use of strategies for an oriented immobilization of capture molecules^19–23,27,28^, the search for alternatives to NC as the analytical strip material^24–28^ and the use of more sensitive reporters^15^, among other.

Here we show that the material and molecular parts of a conventional LFA cartridge can be redesigned to explore the affinity of CBM for cellulose as a means of anchoring capture antibodies. Firstly, cellulose was incorporated on standard NC strips by promoting the in situ regeneration of cellulose fibers over the test and control lines areas of NC analytical strips using NMMO as solvent (Fig. 2). SEM images show that this layer of cellulose fibers sits on top of the NC. When fluid wicks through these zones, part of it will travel across the top cellulose layer and part through the underlying NC membrane (Fig. 2). Our results show that this layer of cellulose fibers provides an anchoring point for biomolecules and contributes to increase its number close to the surface of the strips, and hence the intensity of the signals generated (Fig. 3). The use of an analytical strip made entirely of cellulose paper, as proposed earlier^26–28^, could have been considered as opposed to the layering strategy used. However, by restricting the cellulose fibers to the test and control zones of the analytical strip, we can still take advantage of the excellent and superior flow properties of NC.

ZZ-CBM3 fusions were then used at the cellulose zone of the test line to anchor capture antibodies (Fig. 1d). This new LFA architecture was applied to the fluorescence-based detection of Cys-C in urine samples using an antibody sandwich assay. The fusion acted as an interface molecule between the cellulose layer and the Fc portion of capture antibodies. The planar carbohydrate-binding interface of CBM3 binds to the surface of the cellulose microfibrils while the double Z domain anchors the capture antibody with an orientation that is more favorable to an effective capture of analytes. The layering of cellulose over NC not only provided a cellulose area for interaction with the CBM3 moiety but also contributed to increase the intensity of the fluorescence signals generated at the lines (Fig. 4). We further show that the interference of full-length detection antibodies with the ZZ-CBM3 fusion, which had already been identified by Yang et al*.* as a potential problem when using ZZ fusions in immunoassays^27^, can be overcome by resorting to Fab fragments that lack the Fc portion (Fig. 5). This makes it then possible to use a single ZZ-CBM fusion to anchor virtually any Fc-containing immunoglobulin in LFA. This provides a higher degree of flexibility compared to the solution proposed by Elter et al*.*, which relied on the design of specific CBM-antibody fusions for each analyte system^28^. The downside of our approach, however, is that Fab fragments must be procured or prepared from full length antibodies.

The concentration–response relationship in the 0–10 ng/mL Cys-C concentration range obtained with the new LFA architecture displayed a linear behavior (Fig. 7) and an inter assay CoV of 0.72%. This range is compatible with the clinical diagnostic range for Cys-C (5–120 ng/mL in healthy patients, > 250 ng/mL in patients with kidney disease), as long as proper sample dilution is performed. Finally, the new LFA was used to detect Cys-C in mock urine samples that are representative of normal (100 ng/mL) and kidney tubular disease (4000 ng/mL) patients (Fig. 8).

Overall, we demonstrate a new LFA architecture that combines NC strips with layered cellulose, ZZ-CBM3 fusions and fluorescently labeled Fab fragments. The new LFA constitutes an addition to Cys-C LFA assays reported in the literature (see Table 1), which exclusively rely on the use of anti-Cys-Ccapture antibodies physically adsorbed on NC. We recognize that there is an added complexity to our design that increases costs since an extra deposition (of cellulose), a ZZ-CBM3 fusion and antibody fragments are required. Nevertheless, our results show that there are gains in sensitivity that could be translated into savings in the amount of capture antibodies used. While Cys-C was used as the model system, we believe that this design is worth exploring in the context of the detection of many other analytes.

**Table 1:** Comparison of reported LFAs used for cystatin C detection. A cystatin C ELISA is also shown.

LOD (ng/mL)	Description of system	References
nd	LFA with an analytical NC strip with layered cellulose, anti-cystatin C capture antibodies anchored via ZZ-CBM3 and detection with anti-cystatin C Fab fragments labeled with Alexa Fluor-647	This work
23	LFA with an analytical NC strip, anti-cystatin C capture antibodies adsorbed on test line and detection with full-length antibodies labeled with Alexa Fluor-647	^53^
13	LFA with an analytical NC strip, anti-cystatin C capture antibodies adsorbed on test line and detection with CysC specific aptamer labeled with Alexa Fluor-647	^54^
0.61	LFA with an analytical NC strip, anti-cystatin C capture antibodies adsorbed on test line and colorimetric detection with full-length anti-cystatin C antibodies labeled with plasmonic-fluorescent labels	^55^
0.24	LFA with an analytical NC strip, anti-cystatin C capture antibodies adsorbed on test line and fluorescence detection with full-length anti-cystatin C antibodies labeled with plasmonic-fluorescent labels	^55^
0.50	Sandwich ELISA with anti-cystatin C capture antibodies and colorimetric detection with full-length anti-cystatin C antibodies labeled with horseradish peroxidase	^43^

## Materials and methods

### Materials

Nitrocellulose membranes (HiFlow135) were procured from Merck Millipore (Bedford, MA, USA). Sample and conjugation pad (CF-4) and absorbent pad (CF6) were obtained from Cytiva, UK. The anti-Cys-C monoclonal antibodies (Cyst24 and Cyst28) and the recombinant protein Cys-C (8CY5) were obtained from Hytest Ltd, Finland. Fab2 fragments of the anti-Cys-C antibody (Cyst28) were generated using the Fab2 Fragmentation Kits (Cat. # 786-274, 786-864) from G-Biosciences (St. Louis, MO, USA) according to the manufacturer protocol. Biotin-BSA, streptavidin and Alexa Fluor™ 647 NHS Ester were from Thermo Fisher (Waltham, MA, USA), Sephadex G20 column from GE Healthcare (Uppsala, Sweden). PBS (137 mM NaCl, 2.7 mM KCl, 10 mM Na_2_HPO_4_, 1.8 mM KH_2_PO_4_) and PB (75.4 mM Na_2_HPO_4_–7H_2_O, 24.6 mM NaH_2_PO_4_H_2_O) buffers, NaOH, NaHCO_3_, NaN_3_, BSA and Tween-20, N-methylmorpholine N-oxide (NMMO) were purchased from Sigma-Aldrich (St. Louis, MO, USA). CNF (ref. NG01NC0201, 10–20 nm width, 2–3 µm length) were bought from Nanografi Nano Teknoloji, Turkey. Fusions of Carbohydrate Binding Module 3A from *Clostridium thermocellum* and ZZ (ZZ-CBM3, Catalogue number: CZ04981) were obtained from NZYTech, Portugal. Artificial urine (product number NCZ-APS-0014) was from NanoChemazone (Edmonton, Canada).

Test and control lines were dispensed over the analytical strip with an Easy Printer Model LPM-02 from MDI-Advanced Microdevices Pvt. Ltd. (Ambala, India). A portable immunoanalyser (ImageQuant) developed and designed at the Healthcare Technology Innovation Center (IIT, Madras) [Joseph, Shah] was used to evaluate the fluorescence signals generated at the LFA test and control lines (supplementary material S1). ImageQuant uses a laser-based confocal optics system to measure the fluorescence of the test and control lines of the LFA strips. Images are analysed with LabVIEW™ software (National Instruments, Austin, TX, USA) to obtain signal data from test and control line. The system uses intelligent image-analytics techniques that identify the reaction kinematics from a sequence of images, tracks the progress and development of fluorescence at the test and control lines, identifies the stabilization of the reaction and calculates the areas and area ratios of test and control line^56,57^.

### Antibody labeling

The monoclonal anti-Cys-C antibody (Cyst28) and the corresponding Fab fragments were conjugated with a fluorescent organic dye. Alexa Fluor 647 dye possesses a succinimidyl ester that reacts with primary amines on the antibody. Briefly, the detection antibody @ 1 mg/mL in 10 mM phosphate solution (pH 7.4) containing 140 mM NaCl (PBS) was reacted with the organic dye (20 molar excess) dissolved in DMSO for 1 h at room temperature. The fluorescent conjugates were purified using PD-10 desalting columns packed with Sephadex G-25 resin and operated with a spin protocol (centrifugation for 5 min at 1300 × g) as per manufacturer’s instructions. The labelled antibody was collected in 1 × PBS buffer. The synthesized conjugate was mixed with glycerol to a final concentration of 10 µg/mL in PBS buffer pH7.4 and 2% glycerol and stored at − 20 °C until used.

### LFA strip assembly

The LFA strip was assembled by sequentially joining and partially overlapping a sample pad (9.5 mm length), a polyester fiber conjugate membrane (6 mm length), a NC strip (27.8 mm length) and an absorbent pad (13.2 mm length). Layering of cellulose was performed by dispensing a 0.5% (w/w) solution of CNF dissolved in a NMMO solution over the NC strip in the form of lines on the test and control region using the Easy Printer (see supplementary material S1). The suspension was dispensed repeatedly on the same position to increase the concentration of cellulose. The NC strips were dried overnight at room temperature. The sample pad was pretreated with sample pad buffer (PBS), and dried for 1 h at RT. The conjugate pad was immersed in a 0.3 ng/mL solution of antibody-dye conjugate diluted in 100 mM PB buffer with 0.1% Triton, 0.1% BSA, 20% sucrose and subsequently dried for 1 h at 40 °C.

Depending on the experiments, different biomolecules were dispensed over the strips. In general, 0.5 mg/mL solutions of the capture anti-Cys-C antibody, ZZ-CBM3, protein A in 1 × PBS were dispensed over the analytical strips at a rate of 1 µL/cm using the Easy Printer. In some experiments ZZ-CBM3 and capture antibody were conjugated prior to dispensing. This conjugation was performed by incubating 0.5 mg/mL of capture antibody solution with ZZ-CBM3 at a molar ratio of 1:1.5, for 10 min at room temperature. The control system used consisted of lines of biotin-BSA and Alexa-labelled streptavidin. Following dispensing, the analytical strips were kept at 37 °C for 1 h. Finally, the pads and analytical strips were laminated with a partial overlapping of 2 mm and cut with a width of 3.2 mm. The assembled LFA strips were kept at 4 °C until used.

Scanning electron microscopy (SEM) using a FEI (Quanta 200) SEM located at International Centre for Clean Water (ICCW), IIT Madras was used to image cellulose layers over NC. Prior to this analysis, a drop of CNF dissolved in an NMMO solution was deposited over NC samples.

### LFA procedure

Experiments were initially designed to evaluate the feasibility of using cellulose coatings and CBMs in LFAs, which involved dispensing different biomolecules in the test lines of NC and NC + cellulose strips, addition of Alexa-labeled biomolecules to conjugate pads, cartridge assembly and running of samples containing adequate biomolecules. The typical test procedure involved the addition of 75 μL of the appropriate sample to the sample pad of the LFA cartridge under examination, which was subsequently inserted into the ImageQuant analyser. The run button was pressed, and the process was monitored for about 15 min. The signal generated in the NC membrane was scanned by the instrument as a two-dimensional pixel map to quantify the fluorescently labeled biomolecule. The fluorescent pixel map was processed using the NI Lab View software as previously described^56,57^. All quantitative data were assessed with GraphPad Prism 6.0 (GraphPad Software, La Jolla, CA, USA). Fluorescence intensity data were used to calculate the pixel volume of the test, V_T_, and control, V_C_, lines, which correspond to the two-dimensional summation of all pixel intensities within each line^56^. The corresponding mean volume ratio, V_R_, defined as the ratio V_T_/V_C_, was also calculated when required. Assays were performed in triplicate for each sample.

### Cystatin C standards and samples

Calibration curves in the 0–10 ng/mL concentration range were constructed using Cys-C standards prepared in 0.1 M PB with 1% BSA and 0.1% Tween-20. Following LFA analysis (see “Calibration Curves” section above), the mean volume ratio, V_R_, was plotted versus the Cys-C concentration to generate calibration curves. The standard deviation, SD, across triplicates was used to calculate the coefficient of variation (CoV) according to CoV = SD/mean × 100%. Calibration curve data was analysed by linear regression. Mock urine samples were also prepared using artificial urine, which are representative of Cys-C levels in normal (100 ng/mL) and kidney tubular disease (4000 ng/mL) patients. Standard samples with those same concentrations were also prepared in buffer. The samples with normal and abnormal Cys-C concentration were diluted with buffer 1:20 and 1:800, respectively, to bring the concentration down to 5 ng/mL. These diluted samples where then subjected to LFA analysis (see “Detection of cystatin C in mock urine samples” section above).

## Supplementary Information

Supplementary Information.
