# Supplementary material for: Exploring carbohydrate binding module fusions and Fab fragments in a cellulose-based lateral flow immunoassay for detection of cystatin C
Source: Sci Rep. 2022 Mar 31;12:5478. doi: 10.1038/s41598-022-09454-9 (PMC8970072; doi:10.1038/s41598-022-09454-9)
Supplement: Supplementary file 1 — Supplementary Information. [file 41598_2022_9454_MOESM1_ESM.docx]

*Supporting information for*

Exploring carbohydrate binding module fusions and Fab fragments in a cellulose-based lateral flow immunoassay for detection of cystatin C

Satheesh Natarajan^1^, Jayaraj Joseph ^1,2^, Duarte M. F. Prazeres^3,4,*^

1- Healthcare Technology Innovation Centre, Indian Institute of Technology-Madras, Chennai, Tamil Nadu 600113, India

2. Department of Electrical Engineering, Indian Institute of Technology, Chennai, Tamil Nadu 600113, India

3- iBB-Institute for Bioengineering and Biosciences, Department of Bioengineering, Instituto Superior Técnico, Universidade de Lisboa, 1049-001 Lisboa, Portugal

4- Associate Laboratory i4HB—Institute for Health and Bioeconomy at Instituto Superior Técnico, Universidade de Lisboa, Av. Rovisco Pais, 1049-001 Lisboa, Portugal

*corresponding author: D.M.F. Prazeres

e-mail: miguelprazeres@tecnico.ulisboa.pt

Tel: +351-218419133

**This file includes:**

Supplementary info S1

Supplementary Figures S1-S8

**S1. Image-based Quantitative Immunoassay Analyzer (ImageQuant)**

A portable immunoanalyser (ImageQuant) developed and designed at the Healthcare Technology Innovation Center (IIT, Madras) [1,2] was used to evaluate the fluorescence signals generated at the LFA test and control lines (Fig. S1). ImageQuant uses a laser-based confocal optics system to measure the fluorescence of the test and control lines of the LFA strips. Images are analysed with LabVIEW™ software (National Instruments, Austin, TX, USA) to obtain signal data from test and control line. The system uses intelligent image-analytics techniques that identify the reaction kinematics from a sequence of images, tracks the progress and development of fluorescence at the test and control lines, identifies the stabilization of the reaction and calculates the areas and area ratios of test and control line [1,2].


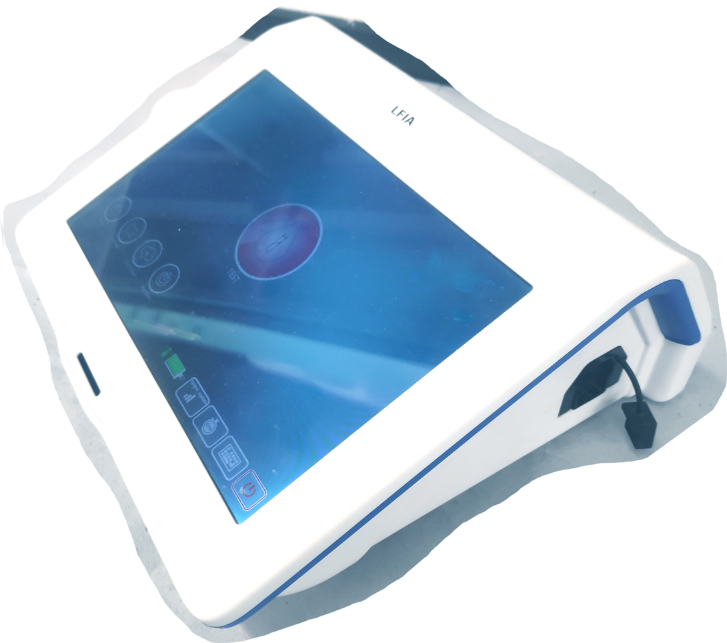


**Figure S1.** Photo of the Image-based Quantitative Immunoassay Analyzer (ImageQuant) used to evaluate the fluorescence signals generated at the LFA test and control lines. ImageQuant was developed at HITC.

**S2. Preparation of solutions of cellulose on NMMO and dispensing over NC strips**

N-methylmorpholine N-oxide (NMMO) can be used to dissolve cellulose by an entirely physical process. The dissolution process includes disintegration of fibers and gradual dissolution. The cellulose’s intermolecular hydrogen bonds are disrupted due to the formation of hydrogen bonds between the anhydrous glucopyranose unit (AGU) of cellulose and the active N–O dipoles and the oxygen groups of NMMO. The new hydrogen bonds contribute to form a strong complex in the homogeneous cellulose solution [3]. The solutions of cellulose in NMMO used in this work were prepared as described in the literature as follows [4]. Firstly, an aqueous solution of NMMO (Sigma-Aldrich) was prepared by mixing 50 g of NMMO in 100 ml of water. Then, 3 g of CNF (ref. NG01NC0201, Nanografi Nano Teknoloji, Turkey) material was added, the mixture was heated at a temperature of 95 °C and the excess water was removed under low pressure (of about 6.7 kPa). The process was continued until a translucid and homogeneous solution of cellulose in NMMO (~0.5 mL) was obtained (Fig. S2a).

Regenerated cellulose materials can be prepared from solutions of cellulose dissolved in NMMO in the form of ﬁbers, ﬁlms, membranes, hydrogels, etc. The process entails the bottom-up self-assembly of cellulose chains in parallel to form ﬁber with highly oriented and crystalline structures [3,5]. In this work we dispensed solutions of dissolved cellulose in NMMO over NC strips to promote the *in-situ* formation of regenerated cellulose fibers. For this, the cellulose solutions prepared as described above (0.5 mL) were combined with 100 mL of Milli-Q water to obtain a solution of dissolved CNF, which was then dispensed over NC (HiFlow135, Merck Millipore) strips in the form of lines on the test and control region using an Easy Printer Model LPM-02 from MDI-Advanced Microdevices Pvt. Ltd. (Ambala, India) and at a rate of 1 µL/cm. The solution was dispensed repeatedly (five times) on the same position to increase the concentration of cellulose. The NC strips were then allowed to dry overnight at room temperature. The width of the cellulose on the NC membrane after drying varied between 2.7-2.9 mm (see Fig. S2b).


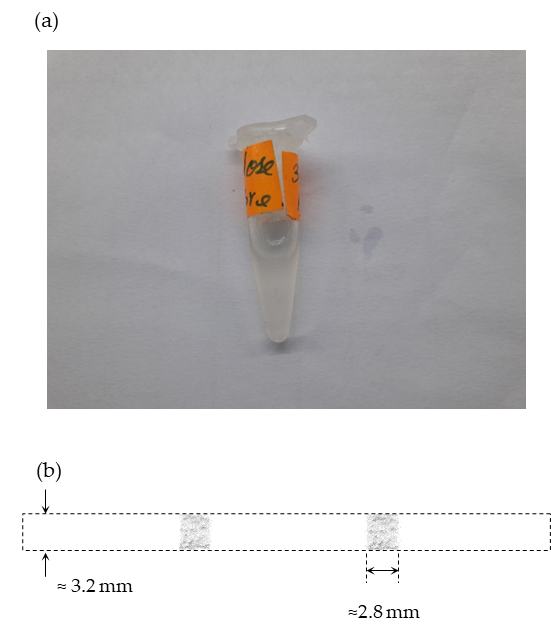


**Figure S2.** (a) Translucid and homogeneous solution of dissolved CNF in NMMO. (b) Photo of a representative NC strip with deposited cellulose. The photo was edited to enhance the contrast between the NC and the cellulose coating.

**S3. Cellulose deposited on NC constitutes an anchor point for ZZ-CBM3 fusions**

A preliminary test was performed to confirm that ZZ-CBM3 fusions bind to the layer of regenerated cellulose deposited on NC via affinity interactions. A cellulose line was dispensed over an NC strip (see S2), which was then laminated as a half strip with an absorbent pad using backing card (Fig. S3a). The capture antibody was labeled with Alexa Fluor™ 647 and then complexed with the ZZ-CBM3 fusion (0.5 mg/mL of labeled antibody, molar ratio of 1.5 antibody to 1 of ZZ-CBM3). The half strips were then dipped in microplate wells containing ~40 𝜇L of solution of this complex and migration by capillarity was allowed to proceed (Fig. S3a). The strips were then removed from the wells and washed gently by submerging 3 times in water. Next, the test line zone was imaged using a fluorescence microscope (Leica DMI3000 B) set up to detect Alexa fluorescence. Results show that cellulose captures the flowing ZZ-CBM:IgG complexes, thus confirming its specificity for the ZZ moiety (Fig. 3b-c).


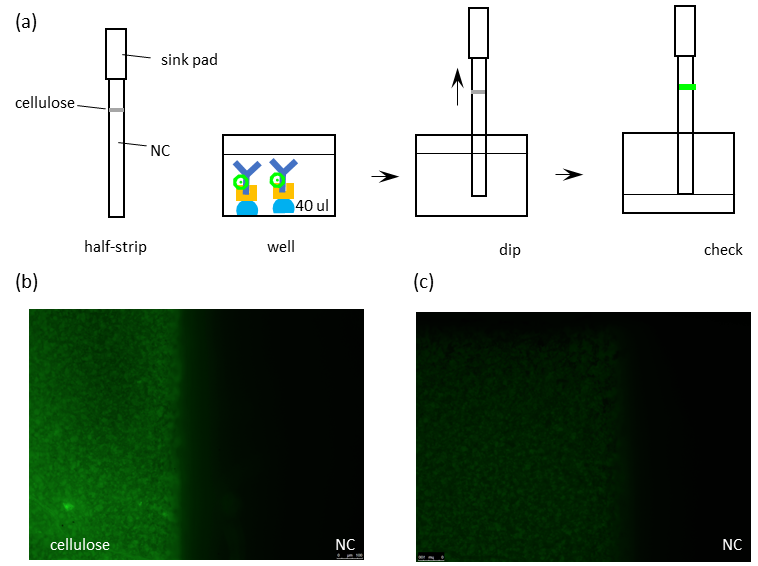


**Figure S3.** Confirmation that regenerated cellulose constitutes an anchor point for ZZ-CBM fusions. (a) schematic overview of tests with half strips. (b) Fluorescence microscope images of the test line zone in the NC strip that was layered with cellulose. The separation between the cellulose region and the NC region is clearly visible. (c) Image of control strip (no cellulose).

**S4. Confirmation of the ability of Alexa-labeled fragments to detect cystatin C**

To check if the Alexa labeled Fab fragments of the detection antibody are able to bind to cystatin C and generate detectable signals, analytical strips of either NC or NC+cellulose were prepared by conventional adsorption of the anti-cystatin C capture antibody in the test line of either NC or NC+cellulose strips. Conjugate pads were then prepared with either Alexa-labeled full detection antibody or with Alexa-labeled Fab fragments of the detection antibody. Following assembly of the LFA cartridges, 5 ng/mL cystatin C samples were run. Results (Fig. S4) show that the detection antibody fragments generate fluorescence signals that are comparable to those obtained with the full detection antibodies.

**Figure S4.** Confirmation of the functionality of Alexa-labeled anti-cystatin C Fab fragments. LFA cartridges with adsorbed anti-cystatin C capture antibodies in test lines and with either Alexa-labeled full detection antibody or with Alexa-labeled Fab fragments were prepared and then run with 5 ng/mL cystatin C samples. Experiments were performed using analytical strips of NC and of NC with a coat of cellulose on the test line. The pixel volume of test lines, *V_T_*, was normalized relatively to the highest pixel volume obtained. The numerical value of the relative *V_T_* is shown in the graph. Experiments were performed in triplicate.

**S5. Schematic illustration of LFA set-ups for obtaining cystatin C calibration curves**


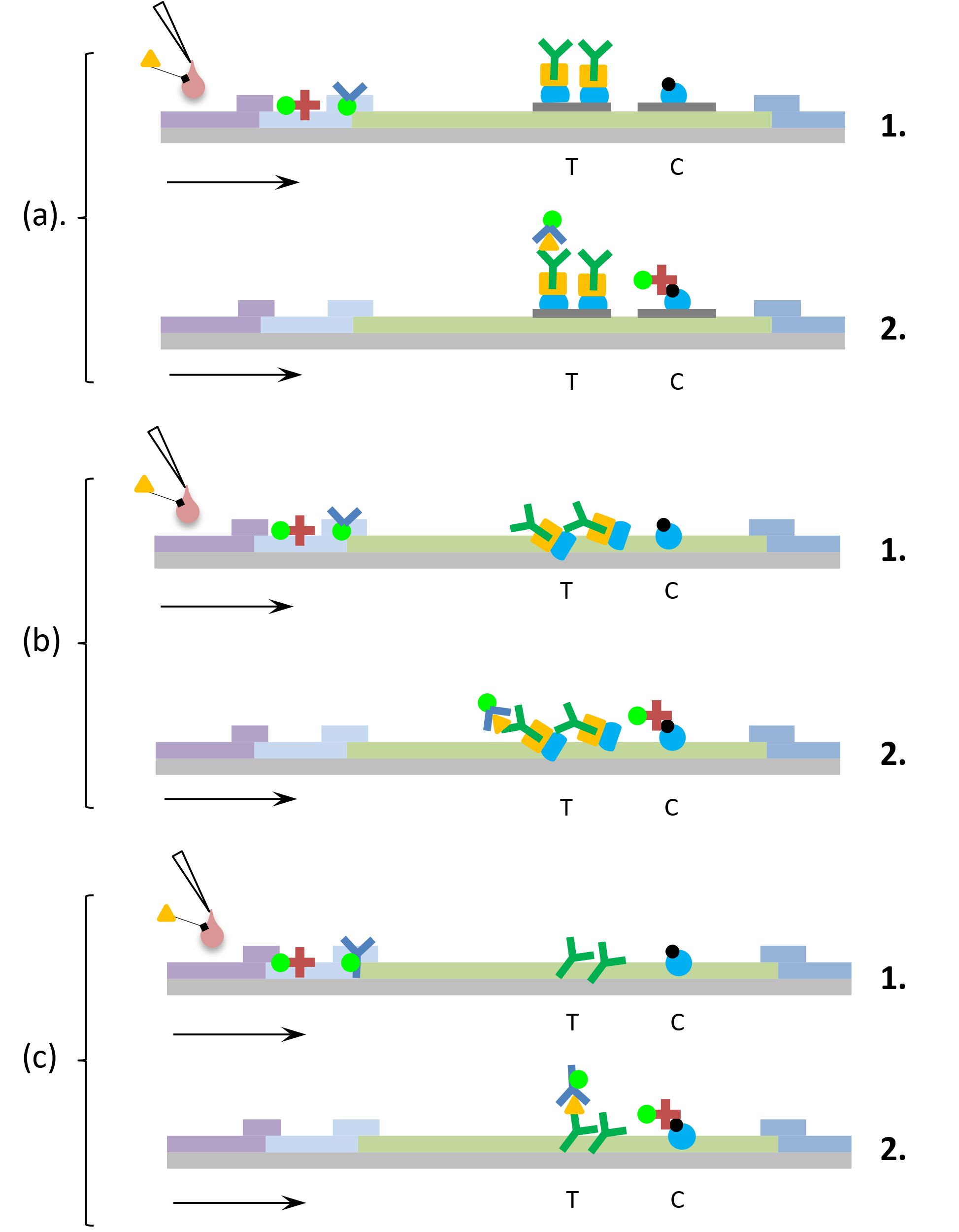


**Figure S5.** Schematic illustration of LFA set-ups for obtaining cystatin C calibration curves. **(a). new architecture:** ZZ-CBM fusions (blue-orange) are conjugated with the capture antibody (green) and dispensed on the test (T) lines of an NC strip with cellulose coated (grey) on the lines; biotin-BSA (blue/black) is dispensed on the control (C) line. Conjugate pads are impregnated with Alexa-streptavidin (red/green) and with Alexa-labelled antibody fragments (blue-green). **(b). intermediate architecture:** ZZ-CBM fusions (blue-orange) are conjugated with the capture antibody (green) and dispensed on the test (T) lines of an NC strip; biotin-BSA (blue/black) is dispensed on the control (C) line. Conjugate pads are impregnated with Alexa-streptavidin (red/green) and with Alexa-labelled antibody fragments (blue-green). **(c). conventional system:** Capture antibodies (green) and biotin-BSA (blue/black) are dispensed on the test (T) and control (C) lines of an NC strip. Conjugate pads are impregnated with Alexa-streptavidin (red/green) and with Alexa-labelled full antibodies (blue-green). 1. Cystatin C (orange) samples with concentrations ranging from 0 to 10 ng/mL are added in the sample pad and 2. captured in the test lines.

**S6. Calibration curves for the detection of cystatin C**

Cystatin C standards with concentrations in the range 0-10 ng/ml were run in LFA cartridges with the new, intermediate and conventional architectures. Experiments were performed in triplicate. Representative fluorescence images of the analytical strips in LFA cartridges are shown in Figure S6.


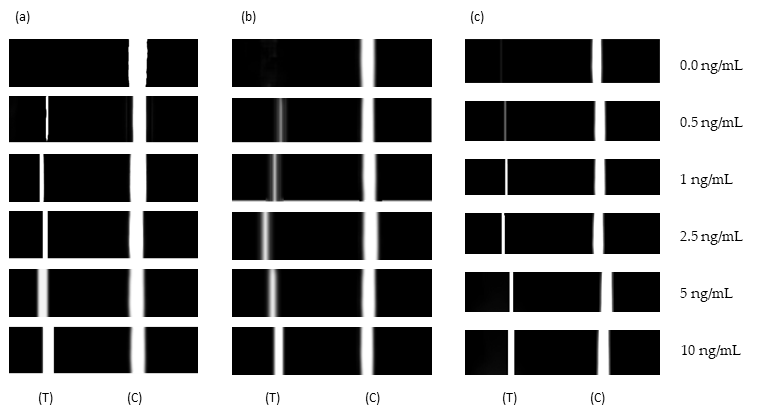


**Figure S6.** Representative black and white fluorescence images of test (T) and control (C) lines of analytical strips in LFA cartridges as captured by the ImageQuant camera. The cystatin C concentrations of samples run in the LFA are displayed next to the photos. (a) New architecture - analytical strip made of NC with layered cellulose, anti-cystatin C capture antibodies anchored via ZZ-CBM3 and detection with Alexa-labeled Fab fragments. (b) Intermediate architecture - analytical strip made of NC, anti-cystatin C capture antibodies anchored via ZZ-CBM3 and detection with Alexa-labeled Fab fragments. (c) Conventional LFA architecture - analytical strip made of NC, anti-cystatin C capture antibodies adsorbed on test line and detection with Alexa-labeled full-length antibodies.

**S7. Detection of cystatin C in mock urine samples**

Experiments were designed to detect cystatin C in mock urine samples prepared in artificial urine that are representative of normal patients (100 ng/mL) and kidney tubular disease patients (4000 ng/mL). Standard samples with the same concentration were also prepared in buffer and used as control. The samples with normal and abnormal cystatin C concentration were diluted with buffer 1:20 and 1:800, respectively, to bring the cystatin C concentration down to 5 ng/mL, which falls within the testing range (0-10 ng/mL). These samples were run in LFA cartridges with the conventional architecture. Experiments were performed in triplicate. Representative fluorescence images of the analytical strips in LFA cartridges are shown in Figure S7.


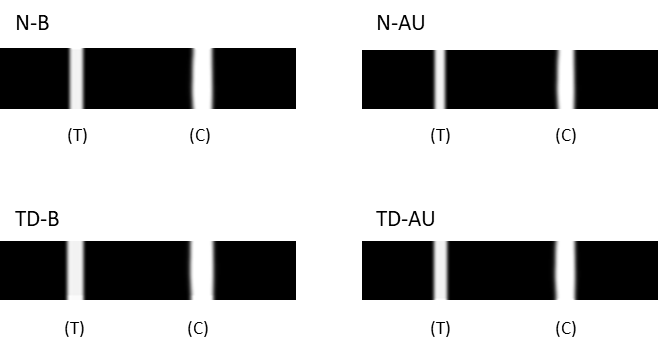


**Figure S7.** Detection of cystatin C in mock urine samples prepared in artificial urine using the new LFA architecture. Black and white fluorescence images of test (T) and control (C) lines of analytical strips in LFA cartridges as captured by the ImageQuant camera. Samples representative of normal (N) patients (100 ng/mL) and kidney tubular disease (TD) patients (4000 ng/mL) were prepared either in artificial urine (AU) or in buffer (B). Analysis was performed using LFA cartridges with the new architecture - analytical strip made of NC with layered cellulose, anti-cystatin C capture antibodies anchored via ZZ-CBM3 and detection with Alexa-labeled Fab fragments.

**References**

1. Joseph, J., Vasan, J.K., Shah, M., Sivaprakasam, M. & Mahajan, L. iQuant™ Analyser: A rapid quantitative immunoassay reader. *Annu. Int. Conf. IEEE Eng. Med. Biol. Soc.* 3732-3736; 10.1109/EMBC.2017.8037668 (2017).
2. Shah, M.I., Rajagopalan, A., Joseph, J. & Sivaprakasam, M. An improved system for quantitative immunoassay measurement in ImageQuant. *2018 IEEE Sensors*, 1-4; 10.1109/ICSENS.2018.8589936 (2018).
3. Tu, H., Zhu, M., Duan, B., Zhang, L. Recent progress in high-strength and robust regenerated cellulose materials. *Adv. Mater.* **33,** 2000682; 10.1002/adma.202000682 (2021).
4. Kulpinski, P. Cellulose nanofibers prepared by the N-methylmorpholine-N-oxide method. *J. App. Polymer Sci.* **98,** 1855–1859; 10.1002/app.22123 (2005).
5. Zhang, S., Chen, C., Duan, C., Hu, H., Li, H., Li, J., Liu, Y., Ma, X., Stavik, J., Ni, Y. Regenerated cellulose by the Lyocell process, a brief review of the process and properties, *BioRes*. **13,** 4577-4592 (2018).
6. Note for Guidance on Validation of Analytical Procedures: Text and Methodology (CPMP/ICH/381/95), ICH Topic Q 2 (R1), 2006, EMEA, London.
